# Outer Retinopathies Associated with COVID-19 Infection: Case Reports and Review of Literature

**DOI:** 10.1155/2024/7227086

**Published:** 2024-03-08

**Authors:** Naima Zaheer, Mohammad O. Tallouzi, N. Ajith Kumar, Sreekanth Sreekantam

**Affiliations:** ^1^Birmingham and Midland Eye Centre, Sandwell & West Birmingham NHS Trust, Birmingham, UK; ^2^Institute of Clinical Sciences, College of Medical and Dental Sciences, University of Birmingham, Birmingham, UK

## Abstract

**Background:**

The coronavirus disease (COVID-19) is a highly contagious disease with profound health implications. It can affect any part of the body with variable severity. Various ophthalmic manifestations of coronavirus disease have been documented. *Case Presentations*. We reported three cases of outer retinopathies associated with COVID-19 infection. All three patients were young females. The first two patients presented within days of COVID-19 infection with complaints of black spots in the eyes. Multimodal retinal imaging showed lesions consistent with acute macular neuroretinopathy. Lesions were bilateral in the first patient and unilateral in the second one. Our third patient presented with blurred vision in one eye, 3 months after a suspected COVID-19 infection. Retinal imaging showed outer retinopathy. Our patients' vision was good and maintained during the follow-up. All three were monitored on observation only, and symptoms and lesions improved with time.

**Conclusion:**

In conclusion, COVID-19-related thromboinflammatory response can result in localized vascular inflammation and hypoperfusion in any of the retinal capillary plexuses or choriocapillaris resulting in ischemia of the corresponding retinal or choroidal layers.

## 1. Introduction

The coronavirus disease 2019 (COVID-19) is a highly contagious disease caused by severe acute respiratory syndrome coronavirus 2 (SARS-CoV-2) infection. It is characterized by an exaggerated inflammatory response and has a wide spectrum of clinical presentations ranging from mild flu-like illness and fever to adult respiratory syndrome, sepsis, coagulopathy, multiple organ disease, and death.

In March 2020, the World Health Organization declared the novel coronavirus outbreak a global pandemic [1]. A wide range of ophthalmological complications of coronavirus disease has been documented [2]. The most frequent ophthalmic complications are those involving the anterior segment (conjunctivitis, keratitis, episcleritis, ocular surface disease, and acute angle closure), followed by posterior segment and neuro-ophthalmological complications (optic neuritis, ischemic optic neuropathy, optic disc swelling, papilledema, extraocular muscle palsies, and orbital compartment syndrome) [2]. Posterior segment complications can affect macrovascular and microvascular circulations leading to retinal venous and arterial occlusions, retinal haemorrhages, and cotton wool spots (CWS) [3, 4]. Retinal vascular abnormalities seem to be evolving as signature ophthalmic manifestations of COVID-19. In a review article, a significant eightfold increase in the prevalence of retinal microvasculopathy was observed in COVID-19 patients [5]. Few case reports also highlighted ocular manifestations as the first sign of COVID-19 infection [6].

In this paper, we are reporting three interesting cases of outer retinopathies associated with COVID-19 infection.

## 2. Case Presentations

### 2.1. Case 1

An 18-year-old Caucasian female presented in the eye casualty with complaints of black spots in both eyes. She was unwell for 3-4 days with fever and fatigue. Her nasopharyngeal swab tested positive for COVID-19. Her visual symptoms (of black spots) started after 2 days of being positive for COVID-19. She was a known asthmatic and was on oral contraceptive pills for a year.

**Figure 1:**
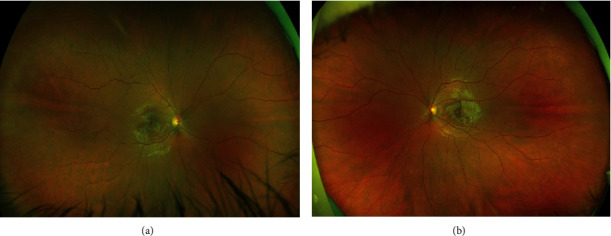
Case 1: wide-field retinal images—obtained by Optos: (a) right eye and (b) left eye.

**Figure 2:**
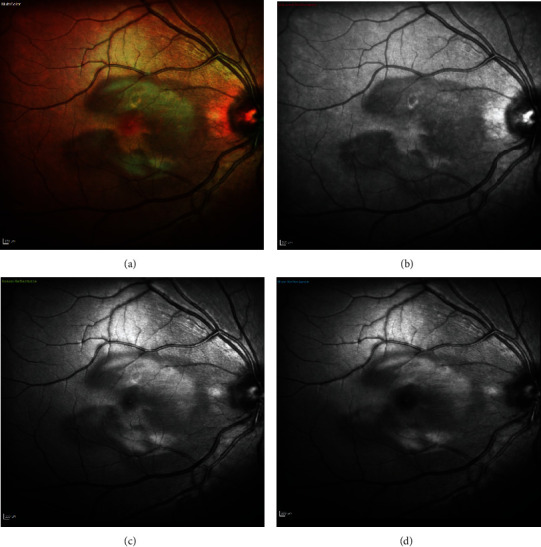
Case 1: multispectral fundus imaging of the right eye showing lesions of acute macular neuroretinopathy—(a) multicolor, (b) with infrared reflectance (IR -820 nm), (c) with green reflectance (GR -515 nm), and (d) with blue reflectance (BR -488 nm).

**Figure 3:**
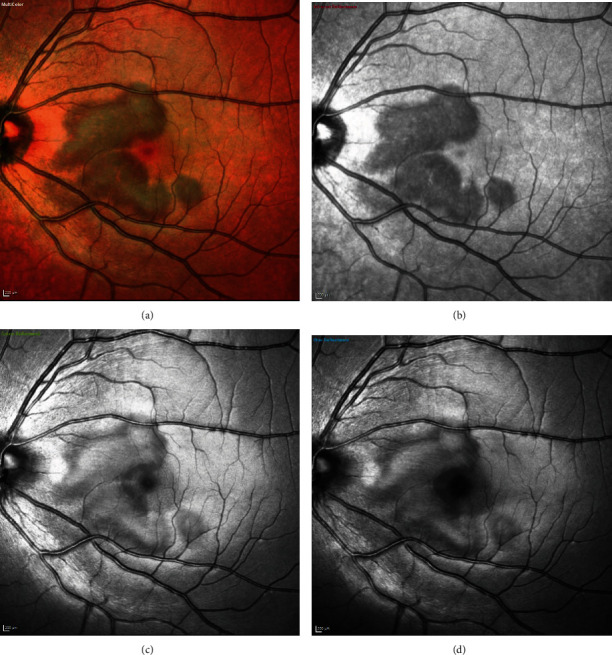
Case 1: multispectral fundus imaging of the left eye showing lesions of acute macular neuroretinopathy—(a) multicolor, (b) with infrared reflectance (IR -820 nm), (c) with green reflectance (GR -515 nm), and (d) with blue reflectance (BR -488 nm).

**Figure 4:**
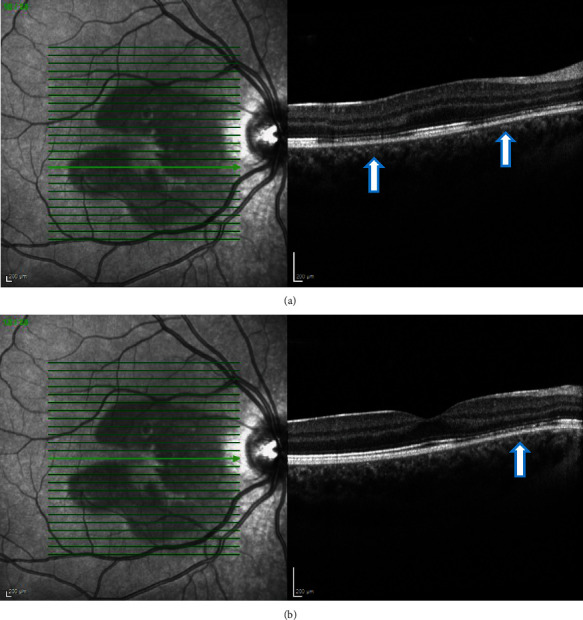
(a, b) Case 1: spectral-domain OCT of the right eye showing focal areas of hyporeflectivity of the external limiting membrane, the cone ellipsoid zone, and the phagosome zone of retinal pigment epithelium.

**Figure 5:**
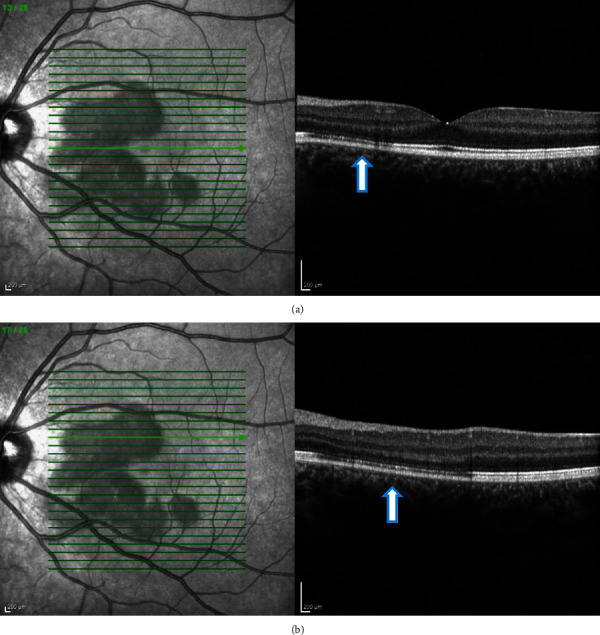
(a, b) Case 1: spectral-domain OCT of the left eye showing focal areas of hyporeflectivity of the external limiting membrane, the cone ellipsoid zone, and the phagosome zone of retinal pigment epithelium.

**Figure 6:**
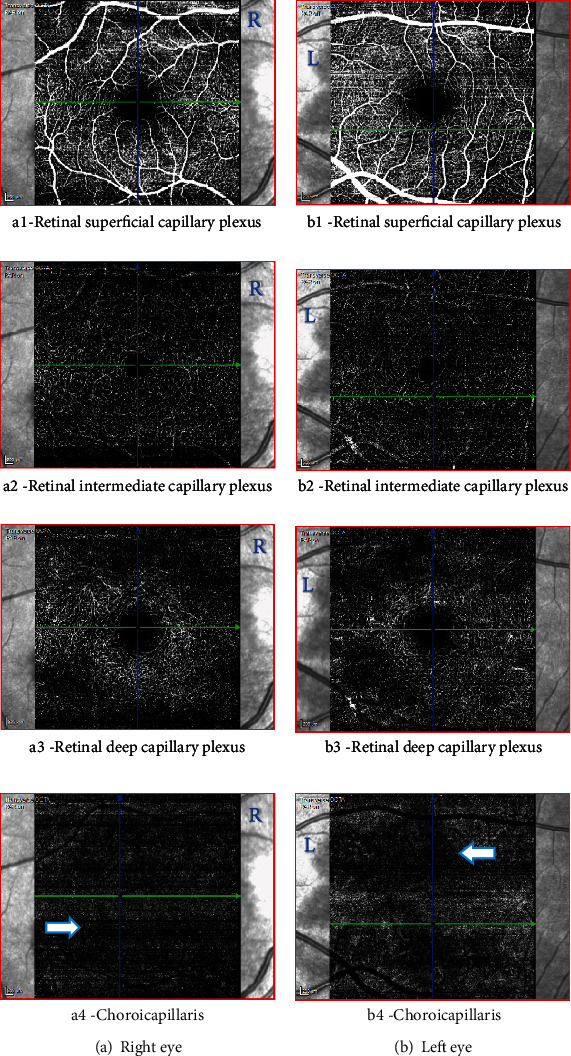
Case 1: optical coherence tomography angiography (OCT-A) of the right and the left eye, showing patches of reduced flow signal in the choriocapillaris.

On examination, visual acuity (using Snellen's chart) was 6/4 in both eyes. There were no signs of the anterior chamber or vitreous inflammation. Fundus examination was unremarkable in both eyes (Figure 1). Multispectral fundus imaging showed well-defined pale lesions around the fovea in both eyes (Figures 2 and 3). Spectral-domain optical coherence tomography (SD-OCT) demonstrated focal areas of hyporeflectivity of the external limiting membrane (ELM), the cone ellipsoid zone, and the phagosome zone of retinal pigment epithelium (RPE) (Figures 4 and 5), correlating with the lesions on multispectral imaging. Optical coherence tomography angiography (OCT-A) showed patches of reduced flow signal in the choriocapillaris (Figure 6). Humphrey's visual fields 10-2 and 24-2 showed bilateral areas of reduced sensitivity corresponding with the lesions. On Multifocal electroretinogram (mf-ERG) areas of reduced amplitude and delayed P1 peak time were observed in both eyes. These changes were more significant nasally and were more significant in the left eye as compared to the right eye. Her blood tests for the inflammatory screen (baseline blood tests including full blood count, erythrocyte sedimentation rate, C-reactive protein, renal and liver function tests, haemoglobin A1c; serum levels of electrolytes, calcium, glucose, angiotensin-converting enzyme, immunoglobulins (IgG, IgM, and IgA), complement (C3 and C4), rheumatoid factor; and autoantibodies including antinuclear antibody, antineutrophilic cytoplasmic antibody, extractable nuclear antibodies, and cardiolipin antibody profile) were negative.

**Figure 7:**
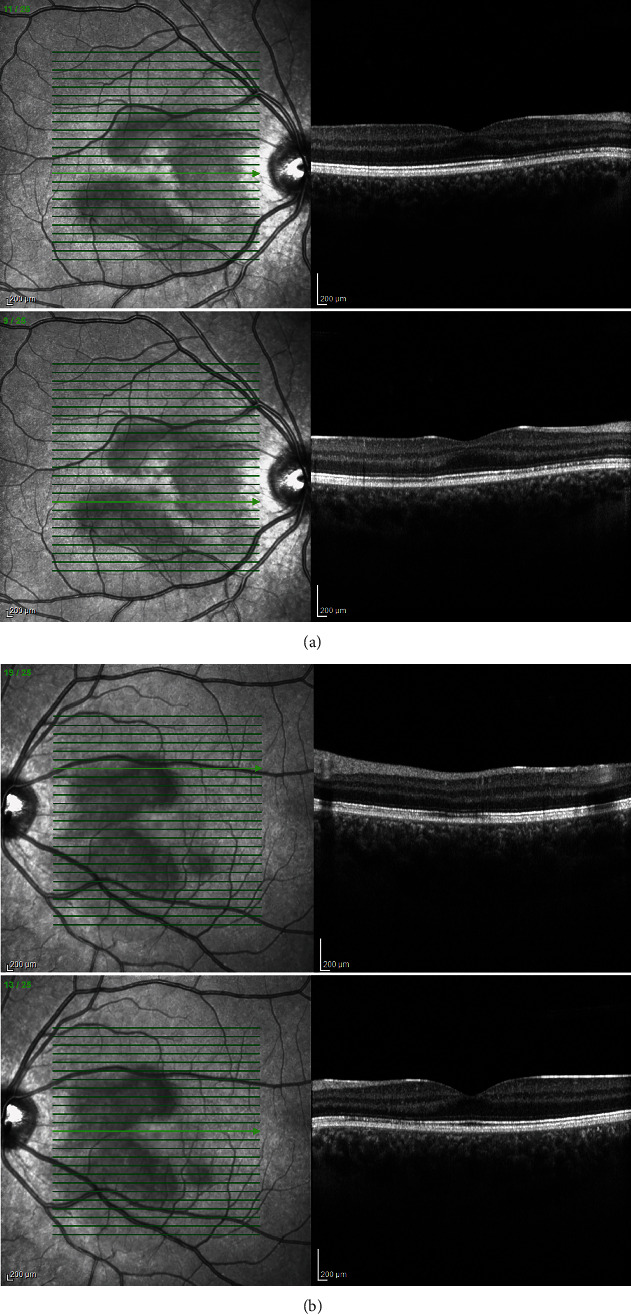
(a, b) Case 1: spectral-domain OCT after 2 months showing restoration of outer retinal layers in the (a) right and (b) left eye.

Diagnosis of acute macular neuroretinopathy (AMNR) possibly secondary to COVID-19 infection was considered. She was kept under close monitoring, and at 2-3 months, the lesions started improving (Figure 7) along with improvement in the visual fields.

### 2.2. Case 2

A 17-year-old Caucasian female presented in the eye casualty with complaints of a black patch in the right eye for 10 days. Initially, she noticed black patches in both eyes. After 1 week, the symptoms improved in the left eye, but the right eye had a persistent patch. Her visual symptoms started after one day of being positive for COVID-19. She had a history of hay fever and eczema and was on oral contraceptive pills for over a year.

**Figure 8:**
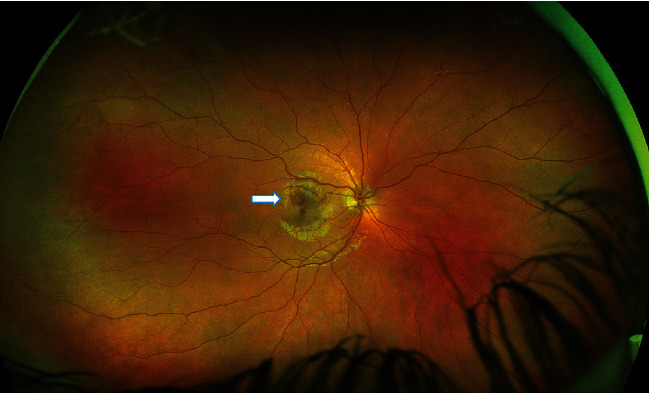
Case 2: wide-field retinal image of the right eye—obtained by Optos—showing a dark spot near the fovea.

**Figure 9:**
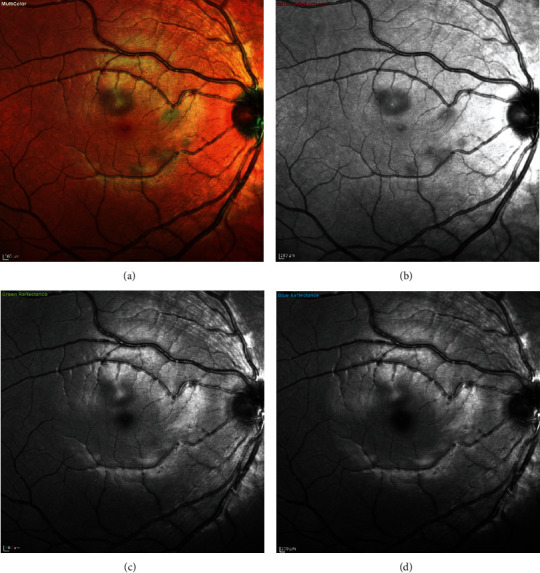
Case 2: multispectral fundus imaging of the right eye showing lesions of acute macular neuroretinopathy—(a) multicolor, (b) with infrared reflectance (IR -820 nm), (c) with green reflectance (GR -515 nm), and (d) with blue reflectance (BR -488 nm).

**Figure 10:**
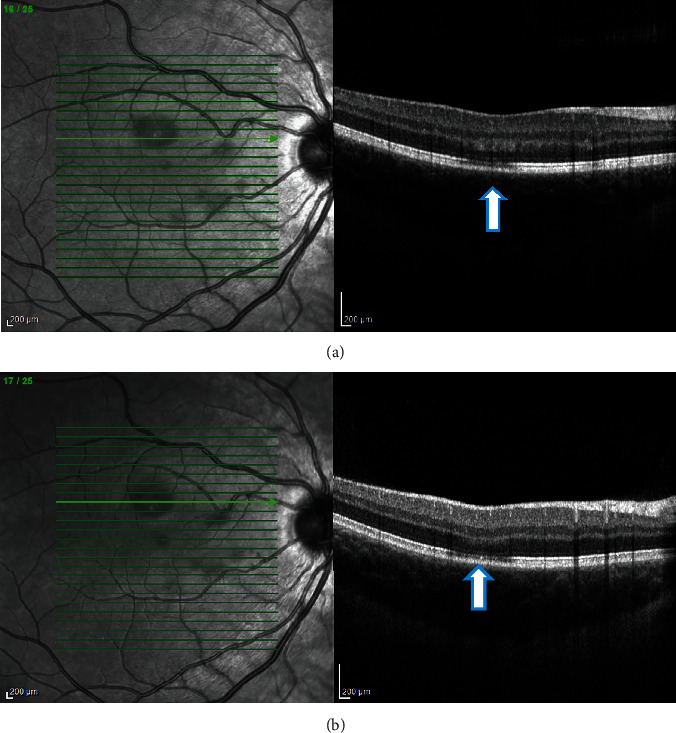
(a, b) Case 2: spectral-domain OCT of the right eye showing a patch of hyporeflectivity of the external limiting membrane, the cone ellipsoid zone, and the phagosome zone of retinal pigment epithelium.

**Figure 11:**
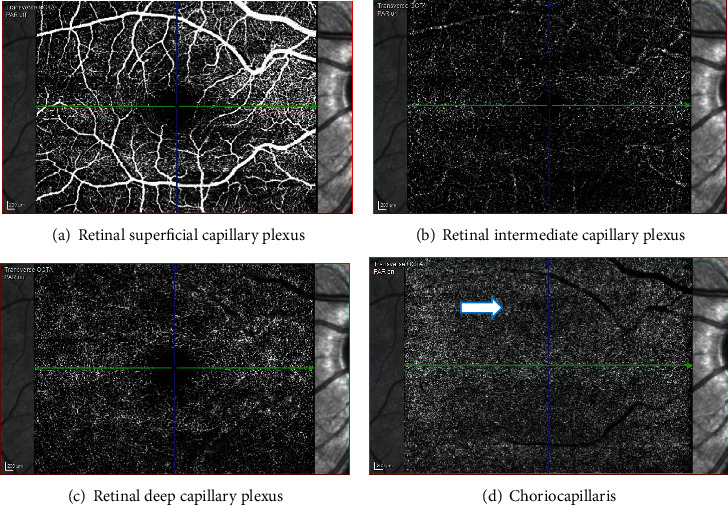
Case 2: optical coherence tomography angiography (OCT-A) of the right eye showing a small patch of reduced flow signal in the choriocapillaris.

**Figure 12:**
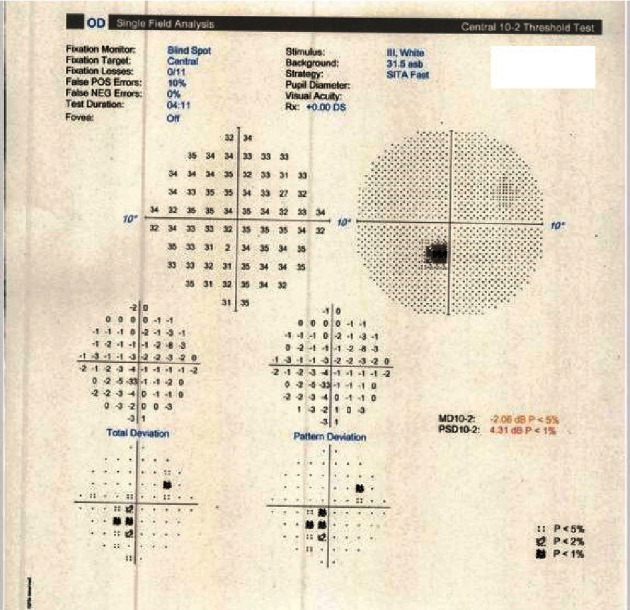
Case 2: Humphrey's visual field 10-2 showing visual field defect in the right eye.

On examination, visual acuity (using Snellen's chart) was 6/5 in both eyes. There were no signs of the anterior chamber or vitreous inflammation. Fundus examination showed a dark spot in the right eye near the fovea (Figure 8). Multispectral fundus imaging highlighted a well-defined pale lesion near the fovea in the right eye (Figure 9). Spectral-domain OCT demonstrated a patch of hyporeflectivity of the ELM, the cone ellipsoid zone, and the phagosome zone of RPE (Figure 10) correlating with the lesion on multicolor imaging. Optical coherence tomography angiography showed a small patch of reduced flow signal in the choriocapillaris (Figure 11). Humphrey's visual field 10-2 (Figure 12) showed an area of reduced sensitivity in the right eye corresponding with the lesion. Her blood tests for the inflammatory screen (the same tests as mentioned earlier in the first case) were negative.

Diagnosis of AMNR possibly secondary to COVID-19 infection was considered. She was kept under close monitoring, and after 2 months, the lesions started improving along with improvement in visual fields.

### 2.3. Case 3

A 14-year-old Chinese girl presented to the eye casualty with complaints of foggy vision in her left eye for one week. There was no associated photopsia or floaters. Approximately three months back, she suffered from a viral illness and COVID-19 symptoms, along with a loss of sense of smell and taste. The nasopharyngeal swab testing was carried out a bit late and came out negative.

**Figure 13:**
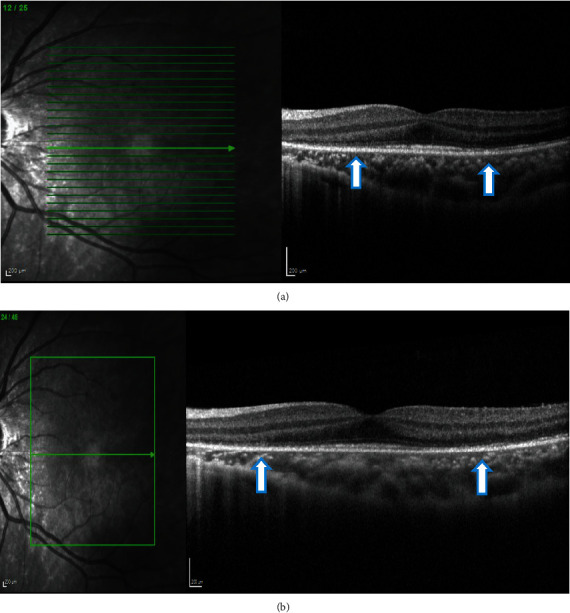
Case 3: (a) spectral-domain OCT and (b) enhanced depth imaging of the left eye at presentation, showing left outer retinopathy, with parafoveal loss of ONL, ELM, cone ellipsoid zone, and RPE phagosome zone.

On examination, visual acuity (using Snellen's chart) was 6/4 and 6/5 in the right and the left eye, respectively. There were no signs of the anterior chamber or vitreous inflammation in both eyes. Spectral-domain OCT showed left outer retinopathy, with parafoveal loss of ONL, ELM, cone ellipsoid zone, and RPE phagosome zone (Figure 13). Her blood tests for the inflammatory screen were normal. However, antibodies to SARS-CoV-2 (SARS-CoV-2 Ig Tc Ab) were detected. She had not received any COVID-19 vaccine till that time.

**Figure 14:**
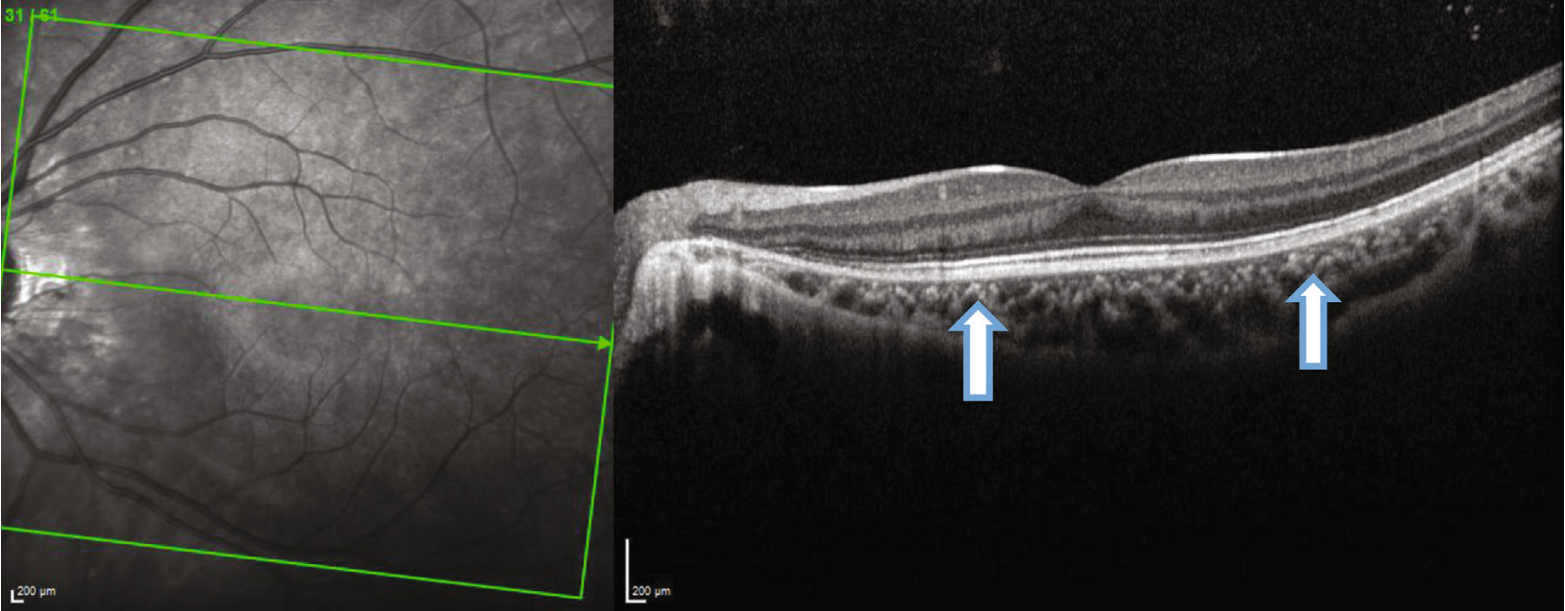
Case 3: spectral-domain OCT of the left eye after 3 months, showing restoration of outer retinal layers.

Diagnosis of outer retinopathy, presumably secondary to COVID-19 infection, was considered, although ocular involvement occurred late after the COVID-19 infection. As her visual acuity was good, she was monitored with observation only, and the outer retinopathy started improving after 6 weeks (Figure 14).

## 3. Discussion

We reported three interesting cases of outer retinopathies and AMNR associated with COVID-19 infection.

For the literature review, we performed a PubMed, Medline, Google Scholar, and Cochrane search using keywords of acute macular neuroretinopathy, COVID-19, and coronavirus infection. We reviewed all the case reports and case series in which AMNR was documented associated with COVID-19 infection.

The coronavirus disease is a highly contagious disease with profound health implications. It can affect any part of the body with variable severity. Various ophthalmic manifestations of coronavirus disease have been documented.

Acute macular neuroretinopathy is a rare retinal disease characterized by reddish brown macular lesions with primary pathology involving hypoperfusion at the level of choriocapillaris affecting outer retinal layers [7].

In our patients, the retinal manifestations were considered secondary to COVID-19 infection. Viral infection has been proposed as the causative mechanism of various outer retinopathies. AMNR has been observed to be preceded by flu-like viral symptoms or febrile illness in approximately 50% of cases [8]. Our two patients were using oral contraceptive pills. The use of oral contraceptive pills has been associated with 35.6% of AMNR patients [8], and oral contraceptives can possibly aggravate the hypercoagulable state caused by the COVID-19 infection.

Regarding the pathogenesis, COVID-19-related ophthalmological manifestations are regarded as a part of COVID-19-related systemic inflammatory response, or secondary to direct viral invasion or a combination of both [9].

One hypothesis is that the retinal and choroidal manifestations are a part of COVID-19-related exaggerated systemic inflammatory response and complement-mediated thrombotic microangiopathies. Various contributory factors have been proposed, including cytokine storm, renin-angiotensin-aldosterone imbalance, the elevation of fibrinogen and D-dimer, and platelet dysfunction [10–12]. All these factors can trigger vasculitis [13], retinal or choroidal vessel endotheliitis, and endothelial cell dysfunction involving venous, arterial, and capillary networks resulting in microvascular and macrovascular thrombotic events [10].

The second attribution is that the retinal and choroidal manifestations can be secondary to direct viral invasion. On immune fluorescence microscopy, there are 2 major proteins of the SARS-CoV-2 virus, spike protein S and nucleocapsid protein N. Protein S is essential for virus internalization and antigenicity [9]. The entry of the virus into the cells is mediated by binding of protein S either to angiotensin-converting enzyme 2 (ACE 2), a specific receptor located on the surface of host cells, or to cluster of differentiation 147 (CD147), a transmembrane glycoprotein. Both ACE 2 receptors and CD147 are expressed in the retina, choroid, and retinal vascular endothelium, facilitating direct virus invasion. SARS-CoV-2 ribonucleic acid (RNA) and proteins S and N have been detected in various layers of the retina, choroid, and optic nerve of the enucleated eyes of COVID-19 patients [9, 14, 15], suggesting that direct virus invasion has a contributory role in the pathogenesis.

It has been observed on OCT-A that macular vascular density measurements in superficial and deep retinal capillary plexus and choriocapillaris were significantly reduced in patients with COVID-19 infection [16–18], which can possibly contribute to increasing the risk of microvascular complications. Our first 2 patients also had localized areas of reduced flow signal in the choriocapillaris contributing to focal ischemia and lesions of outer retinopathy.

COVID-19-related localized retinal or choroidal vessel inflammation results in clinical manifestations in the corresponding layers. COVID-19-related inflammation of the choriocapillaris can cause hypoperfusion and ischemia of the outer retinal layers resulting in AMNR. COVID-19-related inflammation of the retinal intermediate/deep capillary plexus can cause ischemia of the middle retinal layers, INL, and IPL leading to PAMM. In the literature, there are few reported cases of AMNR [19–31] and PAMM [22, 25, 32] associated with COVID-19 infection (Table 1). Goyal et al. [33] and Gascon et al. [34] have reported cases with a combination of both AMNR and PAMM accompanying COVID-19 infection. COVID-19-related inflammation of the retinal superficial capillary plexus can cause ischemia and hyper-reflective bands in the ganglion cell layer (GCL) and nerve fiber layer (NFL) [35, 36].

The incidence of AMNR seems to be increasing since the COVID-19 pandemic and vaccination. In a study from France, Azar et al. studied the cases of AMNR recorded in their hospital in 2019 and 2020 [23]. Eleven cases of AMNR were reported in 2020, during the COVID-19 outbreak. However, only one case of AMNR was documented in 2019, before the pandemic [23].

Regarding the clinical features, all our patients were young females. Our first patient had bilateral ANMR, and others were unilateral. In the literature, the majority of patients with COVID-19-related AMNR were young females with unilateral lesions [23–34], and only a few were bilateral (Table 1) [19–23].

The ocular manifestations can develop after weeks or months after the COVID-19 infection or can be the presenting feature of COVID-19 infection. In our first two patients, the visual symptoms started quite early, within days of the COVID-19 infection. However, our third patient presented late, 3 months after the COVID-19 infection. In literature, retinal signs have been documented varying from 8 to 150 days post-COVID-19 infection (Table 1). It is also known that the COVID-19 virus can persist in tissues for a few months after the infection. Arostegui et al. detected SARS-COV-2 virions in the colon 3 months after infection [37]. Chertow et al. demonstrated the presence of SARS-CoV-2 RNA in multiple anatomic sites including the brain, for up to 230 days postinfection [38]. Acute macular neuroretinopathy has also been reported as the presenting feature of COVID-19 infection [22, 30].

Our patients were systemically well. Despite the significant visual symptoms and characteristic findings on imaging, our patient's visual acuities were good and were maintained during the follow-up. In the majority of the published cases of COVID-19-related PAMM and AMNR, the vision was good.

Our patients were monitored closely and recovered well. In the literature, majority of the COVID-19-related AMNR and PAMM were managed conservatively with observation only, and only one reported case of PAMM secondary to COVID-19 was treated with IV steroids and enoxaparin due to raised D-dimer levels [32].

In conclusion, COVID-19-related thromboinflammatory response can result in localized vascular inflammation and hypoperfusion in any of the retinal capillary plexuses or choriocapillaris resulting in ischemia of the corresponding retinal or choroidal layers.

## Figures and Tables

**Table 1:** Summary of the reported cases of acute macular neuroretinopathy, paracentral acute middle maculopathy, and ischemic retinal microvasculopathies associated with COVID-19 infection.

Review of literature								
Authors	No. of cases	Age	Gender	Duration of ocular signs after COVID-19	Laterality	VA	OCT	Treatment
David and Fivgas [19]	1	22 years	Female	20 days	BL	6/6	AMNR	Observation

Giacuzzo et al. [20]	1	23 years	Female	14 days	BL		AMNR	Observation

Strzalkowski et al. [21]	1	18 years	Female	21 days	BL		AMNR	Observation

Capuano et al. [22]	1	37 years	Female	First sign of COVID-19	BL		AMNR	Observation
	1	27 years Caucasian	Male	First sign of COVID-19	UL		PAMM	Observation

Azar et al. [23]	1	27 years	Female		BL	6/4.8	AMNR	
	1	28 years	Female		BL	6/6	AMNR	
	1	22 years	Female		UL		AMNR	
	1	21 years	Male		UL	6/60	AMNR	

Masjedi et al. [24]	1	29 years	Female	14 days	UL		AMNR	Observation

Virgo and Mohamed [25]	1	32 years Caucasian	Male	16 days	UL		AMNR	
	1	37 years Caucasian	Female pregnant, GA 14 wk	35 days	UL		PAMM	

Komro et al. [26]	1	34 years	Male	64 days	UL		AMNR	Observation

Jalink and Bronkhorst [27]	1	21 years	Female	42 days	UL	6/6	AMNR	Observation
	1	29 years	Female	150 days	UL	6/6	AMNR	Observation

Zamani et al. [28]	1	35 years	Female	6 days	UL	6/6	AMNR and AML	Deceased

Aidar et al. [29]	1	71 years	Female	14 days	UL	6/18	AMNR	Observation

Preti et al. [30]	1	70 years	Male	4 days before COVID-19	UL	6/30	AMNR	Observation

El Matri et al. [31]	1	75 years	Female diabetic	90 days	UL		AMNR	Observation

Padhy et al. [32]	1	19 years Indian	Female	14 days	BL	6/12 & 6/38	PAMM, CWS, and raised D-dimer	I/V steroids and enoxaparin

Goyal et al. [33]	1	32 years	Male	120 days	UL		UL ANMR and BL PAMM	Observation

Gascon et al. [34]	1	53 years	Male	8 days	UL	6/19	AMN, PAMM, deep retinal haemorrhages, and Roth spots	

Ortiz-Egea et al. [35]	1	42 years Caucasian	Male	21 days	UL	6/6	Hyper-reflective band at IPL and GCL	

Marinho et al. [36]	12	25-69 years		11-33 days	BL		Hyper-reflective lesions at IPL, GCL, CWS, and haemorrhages	

## Data Availability

The published papers supporting this case report are from previously reported/published case series/case reports which have been cited in the references.
